# Comparison of standard fusion with a "topping off" system in lumbar spine surgery: a protocol for a randomized controlled trial

**DOI:** 10.1186/1471-2474-12-239

**Published:** 2011-10-18

**Authors:** Jan Siewe, Christina Otto, Peter Knoell, Marco Koriller, Gregor Stein, Thomas Kaulhausen, Peer Eysel, Kourosh Zarghooni, Jeremy Franklin, Rolf Sobottke

**Affiliations:** 1 Department of Orthopaedic and Trauma Surgery, University of Cologne, Kerpener Strasse 62, 50924 Cologne, Germany; 2 Clinical trials centre, BMBF 01KN0706, University of Cologne, Kerpener Strasse 62, 50924 Cologne, Germany; 3 Institute of Medical Statistics, Informatics and Epidemiology, University of Cologne, Kerpener Strasse 62, 50937 Cologne, Germany; 4 Institute for Health Economics and Clinical Epidemiology, University of Cologne, Gleuler Strasse 176-178, 50935 Cologne, Germany

## Abstract

**Background:**

Fusion of lumbar spine segments is a well-established therapy for many pathologies. The procedure changes the biomechanics of the spine. Initial clinical benefits may be outweighed by ensuing damage to the adjacent segments. Various surgical devices and techniques have been developed to prevent this deterioration. "Topping off" systems combine rigid fusion with a flexible pedicle screw system to prevent adjacent segment disease (ASD). To date, there is no convincing evidence that these devices provide any patient benefits.

**Methods/Design:**

The study is designed as a randomized, therapy-controlled trial in a clinical care setting at a university hospital. Patients presenting to the outpatient clinic with degenerative disc disease or spondylolisthesis will be assessed against study inclusion and exclusion criteria. After randomization, the control group will undergo conventional fusion. The intervention group will undergo fusion with a supplemental flexible pedicle screw system to protect the adjacent segment ("topping off").

Follow-up examination will take place immediately after treatment during hospital stay, after 6 weeks, and then after 6, 12, 24 and 36 months. Subsequently, ongoing assessments will be performed annually.

Outcome measurements will include quality of life and pain assessments using questionnaires (SF-36™, ODI, COMI). In addition, clinical and radiologic ASD, work-related disability, and duration of work disability will be assessed. Inpatient and 6-month mortality, surgery-related data (e.g., intraoperative complications, blood loss, length of incision, surgical duration), postoperative complications, adverse events, and serious adverse events will be documented and monitored throughout the study. Cost-effectiveness analysis will also be provided.

**Discussion:**

New hybrid systems might improve the outcome of lumbar spine fusion. To date, there is no convincing published data on effectiveness or safety of these topping off systems. High quality data is required to evaluate the benefits and drawbacks of topping off devices. If only because these devices are quite expensive compared to conventional fusion implants, nonessential use should be avoided. In fact, these high costs necessitate efforts by health care providers to evaluate the effects of these implants. Randomized clinical trials are highly recommended to evaluate the benefits or harm to the patient.

**Trial Registration:**

ClinicalTrials.gov: NCT01224379

## Background

Some controversy remains regarding fusion procedures of the lumbar spine. There is evidence that lumbar spine fusion is more efficient than common nonsurgical treatment [1]. On the other hand, more recent research suggests that primary spinal fusion might not be more beneficial than intensive rehabilitation [2]. Nonetheless, fusion is a well-established surgical treatment for various pathologies of the lumbar spine, and is frequently used for patients with chronic low back pain.

Spinal fusion changes the biomechanics of the spine, e.g., by creating increased motion in the segments adjacent to the fused level. Initial clinical benefits from fusion might be outweighed by the adjacent segment disease (ASD) that ensues. It is important to distinguish between adjacent segment degeneration, which is a radiological term and does not lead to clinical symptoms, and adjacent segment disease. Adjacent segment disease is the clinical presence of symptoms that correlates to degenerative disease seen radiographically adjacent to the index level [3].

As a result, disc arthroplasty and dynamic stabilization techniques have evolved with the hope that the technology can prevent this degeneration [4]. One of the most rapidly evolving techniques in spinal surgery is posterior dynamic stabilization (PDS). Over the past few years, the industry has developed various types of flexible pedicle screw systems, including the "topping off" technique (a rigid system with an attached flexible system for the adjacent segment). Other surgeons perform procedures including a monosegmental rigid fusion plus an interspinous spacer superiorly [5]. To simplify the discussion of PDS devices, Khoueir et al. [6] classified them into:

1. Interspinous spacer devices,

2. Pedicle screw/rod-based devices, and

3. Total facet replacement systems.

The concept is to maintain or restore intervertebral motion in a controlled fashion, whether by restricting extremes of spinal movement or by dampening the kinetic energy involved in motion. The goal of these implantable devices is to mimic the behavior of the healthy spinal column [6,7]. Companies have developed various types of flexible pedicle screw systems (e.g. Surgicraft's Graf ligament, Bioflex's Spring rod, Dynesys^®^, AccuFlex™, Medtronic's Peek rod, Isobar^®^) and topping off systems (e.g. DTO™, DSS™, CosmicMIA™). Topping off has been reported to provide dynamic stabilization along with a consequent reduction of degeneration in the adjacent segment [8,9]. Maserati et al. performed a retrospective analysis of a population after undergoing the topping off procedure. They concluded it was a promising alternative for multilevel fusion with the potential to avert ASD [10]. However, in a recently published, prospective, randomized clinical trial of 60 patients comparing fusion with a hybrid system device versus conventional fusion over 6 years of follow-up, clinical results did not differ between the groups (ODI, VAS, satisfaction). Although the hybrid fixation showed less progression of degeneration in the adjacent segment, there was a higher rate of implant failure. This study group did not recommend prophylactic dynamic stabilization [11]. Overall, to date there is no convincing evidence that these systems provide any clinical benefit whatsoever.

It is not even clear whether the adjacent degeneration visible radiographically is important for clinical outcome. Fusion of spinal motor segments leads to increased forces acting on adjacent levels and can result in ASD. Adjacent instability is reported even 6 to 12 months post-surgery [4,12]. Average rates vary. In a retrospective study, Cheh et al. identified radiographic ASD in 42.6% of patients (average follow up 7.8 years). Oswestry scores were worse in patients with radiographic ASD. Clinical ASD developed in 43% [4]. Other authors have reported incidence of ASD up to 24% (average follow up 39 months). In that study, instability developed more frequently superior to the fusion [12]. Yang et al. found a significant correlation between clinical outcome and ASD [13]. In a 30-year follow-up comparing patients undergoing various spinal procedures (fusion, discectomy, decompression), Kumar et al. [14] found that the incidence of radiographic changes in levels above the operated region was twice as high after fusion as after the other procedures. In contrast, validated scales and functional testing (e.g. SF-36) showed no significant differences in outcome. The author concluded that radiographic changes do not necessarily lead to functional impairment in all patients following lumbar spine fusion for degenerative disc disease. There is other evidence suggesting that radiologic degeneration of the superior adjacent segment does not correlate with clinical results [15]. In a cohort of 215 patients after lumbar spine arthrodesis, Ghiselli et al. reported an adjacent segment disease rate of 16.5% after 5 years. This rate increased to 30.1% at ten-year follow-up [16]. However, a previous review suggests that there may be a correlation between fusion and the development of adjacent segment degeneration when compared to arthroplasty. This correlation appears even stronger when observing adjacent segment disease, thus underscoring the impact of fusion procedures on adjacent segments [3].

### Objective

Do topping off devices lead to better or at least equivalent clinical outcomes compared to standard fusion? Does topping off prevent development of adjacent instability? Does radiographic ASD correlate with clinical outcome? Does topping off lead to a higher complication rate (e.g., implant failure) than regular fusion?

## Methods and Design

This study is designed as a randomized, parallel-group, therapy-controlled trial in a clinical care setting at a university hospital. Patients presenting to our outpatient clinic with degenerative disc disease or spondylolisthesis will be assessed against study inclusion and exclusion criteria. After informed consent and randomization of patients, surgery will be performed. Follow-up examinations will take place immediately after treatment during hospital stay, and then after another 6 and 24 weeks, for a total study duration of 6 months. Data will be assessed after 12, 24, and 36 months for a supplemental investigation. Further assessments will be performed annually, to account for the possibility that conclusions regarding ASD might not be definitive after 36 months.

Experimental research in this trial will be performed with the approval of the ethics committee of the medical faculty of the University of Cologne under the reference number 10-259. Research carried out in the trial will be in compliance with the Helsinki Declaration [17].

### Participants and recruitment

Patients over 30 years of age presenting to our outpatient clinic with degenerative disc disease or spondylolisthesis and indications for monosegmental lumbar spine fusion are eligible for trial inclusion. Surgery will not be considered until at least 6 months of conservative treatment have been concluded. Patients with spondylolisthesis must respond positively to facet joint injection. Radiologic inclusion criteria are summarized in the appendix.

Prerequisite to inclusion is the presence of radiologic degeneration of the adjacent segment (Pfirrmann grades II-IV in MRI findings [18]) without signs of instability. Definition of radiologic and clinical instability as well as further inclusion criteria are summarized in the appendix.

Study subjects will be approached and recruited by experienced spine surgeons. For the screening procedure, an estimated 150 patients per year will undergo primary monosegmental fusion within the department. Recruitment of approximately 30 patients per year is anticipated.

Patients participating in parallel interventional studies as well as patients with lumbar scoliosis (> 25° cobb angle), spondylolisthesis Meyerding IV, and/or radiologic ASD worse than Fujiwara grade II or Pfirrmann grade IV [18,19] are excluded from this study. Further exclusion criteria are summarized in the appendix.

### Interventions

▪ Patients will receive one of two treatments:

▪ Conventional monosegmental posterior lumbar intervertebral fusion (PLIF)

▪ Hybrid system (PLIF + flexible pedicle screw system above the fusion)

### Control group - conventional PLIF

The control group will receive a monosegmental posterior lumbar spine fusion with an intervertebral cage (PLIF). This is the current, well-established therapy for several pathologies of the lumbar spine (e.g. spondylolisthesis, degenerative disc disease). Thus, the control group will receive the standard of care. This is the only acceptable control/comparison in a trial of this kind. Surgery will be performed using the following devices: Optima ZS™ (Zimmer^® ^Spine Germany GmbH, Kiel, Germany), Wave^© ^Lumbar Cage (Advanced Medical Technologies AG, Nonnweiler, Germany)

### Intervention group - hybrid system

The intervention group will receive a hybrid system with PLIF and a flexible pedicle screw system above the fusion. Surgery will be performed using the following devices: Optima ZS™+ DTO™ (Zimmer^® ^Spine Germany GmbH, Kiel, Germany), Wave^© ^Lumbar Cage (Advanced Medical Technologies AG, Nonnweiler, Germany)

Only skilled spine surgeons (with minimum experience of 30 fusion procedures) will participate in the trial. Intraoperative photo documentation will assist in preventing variations of the procedure (e.g., enlargement of decompression/approach). We will also provide an instructional movie on the procedural standards as well as evaluation of the x-rays.

All patients will receive a surgical drain, to be removed 2 days post-surgery. Both groups of patients will receive physical therapy, beginning on the day after surgery. Patients will be discharged only after sufficient convalescence with unremarkable wound healing. Hospital admission lasting 8-10 days will be necessary. Physical therapy will be performed during the inpatient period. After hospital discharge, physical therapy will be continued under outpatient conditions. This therapy will not be standardized in order to reflect reality.

### Outcome measures and assessments

#### Primary Outcome Measures

The focus of this investigation is on subjective and objective clinical benefits for the patient. Functional outcomes will be evaluated using the SF-36™ score after 6 weeks as well as 6, 12, 24, and 36 months after surgery. The SF-36™ is the most frequently used generic health status measure worldwide. In the past, spine surgery investigations have focused on technical outcomes; however, more recently they have turned to clinical results [20,21]. It is unclear whether radiologic parameters correlate with clinical success; thus, they are poor measures of primary outcome. Recent, important randomly controlled trials (RCTs) in spine surgery have used the SF-36™ and ODI tools to assess clinical outcomes [22,23]. Parameters like pain and walking distance are represented in the SF-36. The development of physical (PCS) and mental health (MCS) component scores has eased interpretation and cross-cultural comparison of the SF-36™. The SF-36™ is a standardized questionnaire to detect patient quality of life corresponding to conditions of health. It yields an 8-scale profile of functional health and well-being scores, as well as psychometrically-based physical and mental health summary measures, and a preference-based health utility index. Thus, the SF-36™ and the patient responses contained within are a good measure of primary endpoint. Points are awarded for individual answers by each patient and added to yield a sum total. The SF-36™ PCS scores of the experimental and control groups will be compared to further quantify patient outcome. Subsequent follow-up will repeat the SF-36™ to ensure assessment for clinical impairment and/or the manifestation of a clinical or radiologic instability. This follow-up will continue for up to 3 years and further after treatment. In cases of impairment, x-ray is medically indicated at any point of time.

#### Secondary Outcome Measures

1. Total, MCS, and 8 individual dimensions and subscales of the SF-36™ (version 4.0) for confirmation and clarification of the primary outcome results.

2. ODI (cross-cultural adaption of the ODI version 2.1 for use with German-speaking patients)[24]: this is one of the condition-specific questionnaires recommended for use with back pain patients. The ODI is a standardized, patient-completed questionnaire which gives a subjective percentage score of level of function (disability) in activities of daily living for those rehabilitating from low back pain. The questionnaire examines perceived level of disability in 10 everyday activities of daily living.

3. COMI questionnaire (version 2008): a patient-oriented, short, multidimensional outcome instrument validated for patients with spinal disorders.

4. X-rays will be taken after 6 weeks, 6, 12, 24, and 36 months. Further assessments will be performed annually. These are common intervals after surgical implant procedures not requiring extraneous radiation. At the point in time where adjacent instability is identified radiographically, we will reevaluate and compare the clinical outcome. Criteria for radiologic ASD as well as adjacent instability are listed in the appendix (definition of radiologic instability and Weiner's classification). Pre- and postoperative x-rays must be compared to assess the Weiner's classification score. X-rays will be evaluated by experienced radiologists.

5. Clinical ASD will be assessed (see appendix for the definition).

6. Work-related function/disability at the time of surgery and after surgery, plus the duration until return to work. At each visit, patients will be interviewed about their level of work-related function/disability as well as the time point when/if they return to employment.

7. Inpatient mortality and mortality at 6 months.

8. Surgery-related data (blood loss, length of incision, duration of surgery) are taken from the surgical report.

9. Intraoperative and postoperative complication rates (e.g. implant failure)

10. Adverse events (AE) and serious adverse events (SAE) will be documented and monitored throughout the study.

### Sample size

Our target recruitment is 30 patients. We assume a loss of follow-up of 10%, so that 27 patients should be available for the final analysis. The normalized form of the SF-36™ PCS (mean 50, standard deviation 10) is assumed. With this number of patients, using a two-sided t-test, a normalized PCS score difference of 11.2 or more could be detected with a power of 80% and alpha = 0.05.

### Randomization

The randomization of patients into intervention and control groups will be performed using blocks of randomly variable length in order to maintain balance while preventing predictability of allocation. Randomization is stratified by surgeon. A container with the sequentially numbered, sealed envelopes is stored in a locked cupboard, which only the investigator(s) can open. The random allocation sequence and the sealed envelopes will be generated by the Institute of Medical Statistics, Informatics, and Epidemiology of the university conducting the trial. Enrollment and randomization will be executed by the investigator.

### Data analysis

The PCS and MCS sub-scores and the ODI score are measured repeatedly; thus, areas under the curve (AUC) will be computed. AUCs will be compared between treatment groups using a two-sample t-test.

Time until ASD or adjacent segment instability will be analyzed using the life-table method and compared between treatment groups using the log-rank test.

The primary analysis set is defined according to the intention-to-treat principle, including all randomized patients and interpolating missing longitudinal data. Robustness of results will be investigated by additional analyses based on the per-protocol set. Interim analyses are not foreseen.

### Cost-effectiveness analysis

For our analysis, both a hospital and a societal perspective will be used. Because the surgical treatments to be analyzed do not differ in their DRG-codes, the costs for the hospital will be estimated based on differences in costs of implants used for the surgery and length of hospital stay. Cost-benefit is then expressed as cost per reduced length of stay.

For the societal perspective, additional resource utilization after discharge from hospital will be assessed based on a patient diary. After being discharged from the hospital, the patient will report direct medical costs such as those from physiotherapy, visits to general practitioners or specialists, and surgery-related medication (e.g., analgesics). Additionally, indirect costs from work absences will be recorded. For data on efficacy, quality-adjusted life years (QALY) are estimated by using the SF-36^® ^Health Survey, a generic outcome measure designed to examine a person's perceived health status. An incremental cost-effectiveness ratio in terms of costs per QALY will be calculated.

## Discussion

New hybrid systems might improve the outcome of lumbar spine fusion. To date, however there is no convincing published data regarding these topping off systems. High quality data is required to provide a preliminary impression of the advantages and disadvantages of such devices. If only because topping off devices are quite expensive compared to conventional implants, nonessential use should be avoided. In fact, these high costs necessitate efforts by health care providers to examine the effects of these implants. Randomized clinical trials are highly recommended to evaluate the benefits or harm to patients.

In the intervention group, the approach to the spine will be enlarged compared to that of the monosegmental fusion group. The longer approach itself could bring about a negative outcome compared to that of regular fusion (e.g., due to muscle damage, blood loss, higher risk of infection). As well, implant failure rates can be compared between the groups. The crucial question, however, is the ability to prevent ASD.

Placebo is not an acceptable option for the control group for fundamentally ethical reasons. Not only would such an option deprive the patient of adequate treatment, but also to have a realistic placebo would require an invasive procedure without the implantation of a medical device, thus exposing him/her to significant risks without providing benefit. Furthermore, a "placebo device" as replacement for the respective topping-off system does not exist.

## List of abbreviations

a.p.: anterior-posterior; ASD: Adjacent Segment Degeneration/Disease; COMI: Core Outcome Measure Index; MCS: Mental Health Component Score; MRI: Magnetic Resonance Imaging; NYHA: New York Heart Association; ODI: Oswestry Disability Index; PCS: Physical Health Component Score; PLIF: Posterior Lumbar Intervertebral Fusion; QALY: Quality-adjusted life years; RCT: Randomized Clinical Trial; SF-36™: Short Form Health Survey-36; VAS: Visual Analogue Scale

## Competing interests

The authors declare that they have no competing interests.

## Authors' contributions

JS, CO, TK, KZ, PK and MK prepared the study and will participate in its design and conduction. JF is responsible for statistical design and analysis. RS and PE formulated the study. All authors have revised the manuscript and will participate in conduction of the study. All authors have read and approved the final manuscript.

## Appendix

### Inclusion criteria

• Informed consent

• Legal capacity

• Age ≥ 30 years

• Indication for monosegmental lumbar spine fusion (PLIF or "topping-off ") L2-S1 with osteochondrosis Modic grades I-III [25-27] or spondylolisthesis Meyerding grades I-III.

• Radiologic signs of degeneration in the adjacent segment of the intended fusion without signs of instability

• Definition of adjacent segment degeneration (MRI)

**Figure 1:**
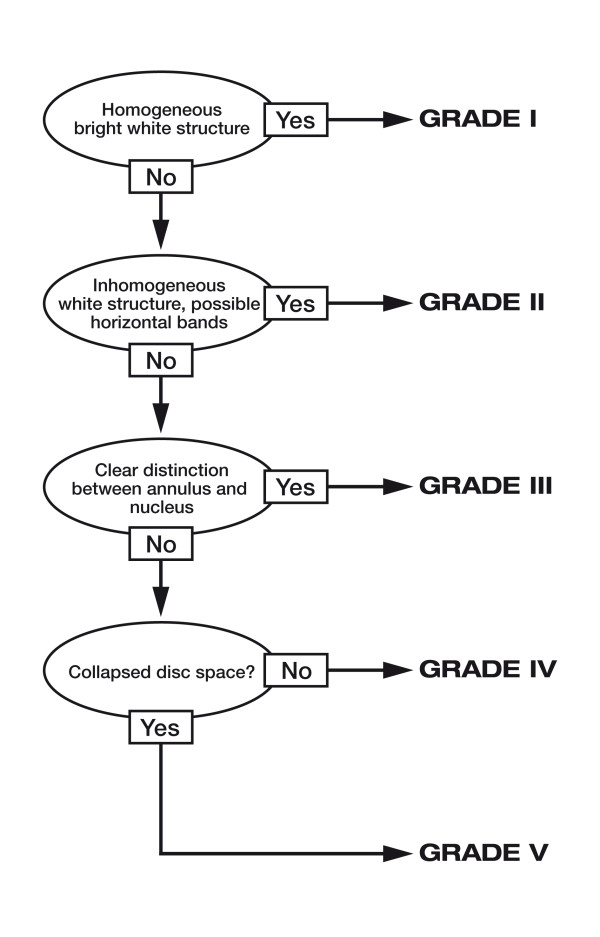
**Decision tree of disc degeneration according to Pfirrmann et al. 2001 **[18].

Pfirrmann [18] grades II-IV (Figure 1)

Definition of radiologic instability (x-ray: a.p. and lateral views, extension and flexion):

1. Spondylolisthesis > 4 mm (anterior or posterior translation) [4]

2. Segmental kyphosis > 10° [4]

3. Rotatory hypermobility > 15° [12]

4. Complete collapse of the disc [4]

5. Lateral translation > 3 mm [12]

6. Disc Wedging > 5° [12]

7. Deterioration in the Weiner's classification of 2 or more grades in the follow-up evaluation [1,27]

Weiner's classification [28]

0 = No disease, defined by normal disc height, no spur formation, no eburnation, and no gas

1 = Mild disease, defined by < 25% disc space narrowing, small spur formation, minimal eburnation, and no gas

2 = Moderate disease, defined by 25%-75% disc space narrowing, moderate spur formation, moderate eburnation, and no gas

3 = advanced disease, defined by > 75% disc space narrowing, large spur formation, marked eburnation, gas present

### Exclusion criteria

• Motor deficit

• Cauda equina syndrome

• Previous surgical intervention of the lumbar spine

• Relevant peripheral neuropathy

• Acute denervation subsequent to radiculopathy

• Scoliosis with Cobb angle greater than 25°

• Spondylolisthesis > Meyerding grade III

• Radiologic signs of degeneration in the adjacent segment of the intended fusion with signs of instability (for definition, see inclusion criteria)

• No radiologic signs of degeneration in the adjacent segment of the intended fusion (for definition, see inclusion criteria)

• Radiologic signs of degeneration in the adjacent segment of the intended fusion with > Fujiwara grade II [19] or > Pfirrmann grade IV [18]

• Signs of instability in any lumbar spine segment other than that undergoing fusion

• General contraindication for elective lumbar spine surgery

• Pathologic fracture

• Osteoporosis with pathologic fracture

• Active systemic infection

• Rheumatic disease

• Disease of bone metabolism (e.g. Paget's disease)

• Bone metastasis

• Local infection focus lumbar spine

• Seizure disorder

• Chronic ischemia Fontaine classification IIb-IV

• Severe heart insufficiency (NYHA III-IV)

• Blood coagulation disorder or blood thinning therapy

• Cortisone intake longer than one month in the 12 months prior to randomization

• Simultaneous participation in another clinical trial in the 30 days before randomization

• Known allergy or intolerance to the implants

• Dependency on investigator

• Lack of familiarity with the German language

• Placement in an institution by governmental or judicial advice

• Absent legal capacity

• Pregnancy

Definition of clinical ASD [12]

1. Symptomatic spinal stenosis

2. Mechanical back pain

3. Sagittal or coronal imbalance

## Pre-publication history

The pre-publication history for this paper can be accessed here:

http://www.biomedcentral.com/1471-2474/12/239/prepub

## References

[B1] Fritzell P Hagg O Wessberg P Nordwall A 2001 Volvo Award Winner in Clinical Studies: Lumbar fusion versus nonsurgical treatment for chronic low back pain: a multicenter randomized controlled trial from the Swedish Lumbar Spine Study Group Spine (Phila Pa 1976) 2001 26 23 2521 2532 discussion 2532-2524 10.1097/00007632-200112010-00002 11725230

[B2] Fairbank J Frost H Wilson-MacDonald J Yu LM Barker K Collins R Randomised controlled trial to compare surgical stabilisation of the lumbar spine with an intensive rehabilitation programme for patients with chronic low back pain: the MRC spine stabilisation trial BMJ 2005 330 7502 1233 10.1136/bmj.38441.620417.8F 15911537 PMC558090

[B3] Harrop JS Youssef JA Maltenfort M Vorwald P Jabbour P Bono CM Goldfarb N Vaccaro AR Hilibrand AS Lumbar adjacent segment degeneration and disease after arthrodesis and total disc arthroplasty Spine (Phila Pa 1976) 2008 33 15 1701 1707 10.1097/BRS.0b013e31817bb956 18594464

[B4] Cheh G Bridwell KH Lenke LG Buchowski JM Daubs MD Kim Y Baldus C Adjacent segment disease followinglumbar/thoracolumbar fusion with pedicle screw instrumentation: a minimum 5-year follow-up Spine (Phila Pa 1976) 2007 32 20 2253 2257 10.1097/BRS.0b013e31814b2d8e 17873819

[B5] Bono CM Vaccaro AR Interspinous process devices in the lumbar spine J Spinal Disord Tech 2007 20 3 255 261 10.1097/BSD.0b013e3180331352 17473649

[B6] Khoueir P Kim KA Wang MY Classification of posterior dynamic stabilization devices Neurosurg Focus 2007 22 1 E3 17608337 10.3171/foc.2007.22.1.3

[B7] Sengupta DK Mulholland RC Fulcrum assisted soft stabilization system: a new concept in the surgical treatment of degenerative low back pain Spine (Phila Pa 1976) 2005 30 9 1019 1029 discussion 1030 10.1097/01.brs.0000160986.39171.4d 15864153

[B8] Caserta S La Maida GA Misaggi B Peroni D Pietrabissa R Raimondi MT Redaelli A Elastic stabilization alone or combined with rigid fusion in spinal surgery: a biomechanical study and clinical experience based on 82 cases Eur Spine J 2002 11 Suppl 2 S192 197 12384744 10.1007/s00586-002-0426-6 PMC3611563

[B9] Kim YS Zhang HY Moon BJ Park KW Ji KY Lee WC Oh KS Ryu GU Kim DH Nitinol spring rod dynamic stabilization system and Nitinol memory loops in surgical treatment for lumbar disc disorders: short-term follow up Neurosurg Focus 2007 22 1 E10 17608331

[B10] Maserati MB Tormenti MJ Panczykowski DM Bonfield CM Gerszten PC The use of a hybrid dynamic stabilization and fusion system in the lumbar spine: preliminary experience Neurosurg Focus 28 6 E2 10.3171/2010.3.FOCUS1055 20568918

[B11] Putzier M Hoff E Tohtz S Gross C Perka C Strube P Dynamic stabilization adjacent to single-level fusion: Part II. No clinical benefit for asymptomatic, initially degenerated adjacent segments after 6 years follow-up Eur Spine J10.1007/s00586-010-1517-4 PMC2997202 20632044

[B12] Aota Y Kumano K Hirabayashi S Postfusion instability at the adjacent segments after rigid pedicle screw fixation for degenerative lumbar spinal disorders J Spinal Disord 1995 8 6 464 473 8605420

[B13] Yang JY Lee JK Song HS The impact of adjacent segment degeneration on the clinical outcome after lumbar spinal fusion Spine (Phila Pa 1976) 2008 33 5 503 507 10.1097/BRS.0b013e3181657dc3 18317193

[B14] Kumar MN Jacquot F Hall H Long-term follow-up of functional outcomes and radiographic changes at adjacent levels following lumbar spine fusion for degenerative disc disease Eur Spine J 2001 10 4 309 313 10.1007/s005860000207 11563616 PMC3611514

[B15] Okuda S Iwasaki M Miyauchi A Aono H Morita M Yamamoto T Risk factors for adjacent segment degeneration after PLIF Spine (Phila Pa 1976) 2004 29 14 1535 1540 10.1097/01.BRS.0000131417.93637.9D 15247575

[B16] Ghiselli G Wang JC Bhatia NN Hsu WK Dawson EG Adjacent segment degeneration in the lumbar spine J Bone Joint Surg Am 2004 86-A 7 1497 1503 15252099 10.2106/00004623-200407000-00020

[B17] Declaration of Helsinki. Ethical principles for medical research involving human subjects J Indian Med Assoc 2009 107 6 403 405 19886379

[B18] Pfirrmann CW Metzdorf A Zanetti M Hodler J Boos N Magnetic resonance classification of lumbar intervertebral disc degeneration Spine (Phila Pa 1976) 2001 26 17 1873 1878 10.1097/00007632-200109010-00011 11568697

[B19] Fujiwara A Tamai K Yamato M An HS Yoshida H Saotome K Kurihashi A The relationship between facet joint osteoarthritis and disc degeneration of the lumbar spine: an MRI study Eur Spine J 1999 8 5 396 401 10.1007/s005860050193 10552323 PMC3611192

[B20] Gibson JN Waddell G Surgery for degenerative lumbar spondylosis: updated Cochrane Review Spine (Phila Pa 1976) 2005 30 20 2312 2320 10.1097/01.brs.0000182315.88558.9c 16227895

[B21] Copay AG Glassman SD Subach BR Berven S Schuler TC Carreon LY Minimum clinically important difference in lumbar spine surgery patients: a choice of methods using the Oswestry Disability Index, Medical Outcomes Study questionnaire Short Form 36, and pain scales Spine J 2008 8 6 968 974 10.1016/j.spinee.2007.11.006 18201937

[B22] Fritzell P Hagg O Jonsson D Nordwall A Cost-effectiveness of lumbar fusion and nonsurgical treatment for chronic low back pain in the Swedish Lumbar Spine Study: a multicenter, randomized, controlled trial from the Swedish Lumbar Spine Study Group Spine (Phila Pa 1976) 2004 29 4 421 434 discussion Z423 10.1097/01.BRS.0000102681.61791.12 15094539

[B23] Wilson-MacDonald J Fairbank J Frost H Yu LM Barker K Collins R Campbell H The MRC spine stabilization trial: surgical methods, outcomes, costs, and complications of surgical stabilization Spine (Phila Pa 1976) 2008 33 21 2334 2340 10.1097/BRS.0b013e318186a8b2 18784631

[B24] Mannion AF Junge A Fairbank JC Dvorak J Grob D Development of a German version of the Oswestry Disability Index. Part 1: cross-cultural adaptation, reliability, and validity Eur Spine J 2006 15 1 55 65 10.1007/s00586-004-0815-0 15856341 PMC3454571

[B25] Modic MT Masaryk TJ Ross JS Carter JR Imaging of degenerative disk disease Radiology 1988 168 1 177 186 3289089 10.1148/radiology.168.1.3289089

[B26] Modic MT Steinberg PM Ross JS Masaryk TJ Carter JR Degenerative disk disease: assessment of changes in vertebral body marrow with MR imaging Radiology 1988 166 1 Pt 1 193 199 3336678 10.1148/radiology.166.1.3336678

[B27] Zhang YH Zhao CQ Jiang LS Chen XD Dai LY Modic changes: a systematic review of the literature Eur Spine J 2008 17 10 1289 1299 10.1007/s00586-008-0758-y 18751740 PMC2556462

[B28] Weiner DK Distell B Studenski S Martinez S Lomasney L Bongiorni D Does radiographic osteoarthritis correlate with flexibility of the lumbar spine? J Am Geriatr Soc 1994 42 3 257 263 8120309 10.1111/j.1532-5415.1994.tb01748.x

